# Psychiatric Morbidity Is Overrepresented in Young Girls at High Risk of Developing Anorexia Nervosa

**DOI:** 10.1002/eat.70104

**Published:** 2026-04-19

**Authors:** Karin Dahlin, Kajsa Järvholm, Jovanna Dahlgren, Elisabet Wentz

**Affiliations:** ^1^ Department of Psychiatry and Neurochemistry Institute of Neuroscience and Physiology, University of Gothenburg Gothenburg Sweden; ^2^ Department of Pediatric Neurology and Psychiatry Sahlgrenska University Hospital, Region Västra Götaland Gothenburg Sweden; ^3^ Department of Psychology Faculty of Social Sciences, Lund University Lund Sweden; ^4^ Department of Pediatrics Institute of Clinical Sciences, University of Gothenburg Gothenburg Sweden; ^5^ Region Västra Götaland, Queen Silvia Children's Hospital, Sahlgrenska University Hospital Gothenburg Sweden; ^6^ Department of Neuropsychiatry Sahlgrenska University Hospital, Region Västra Götaland Gothenburg Sweden

**Keywords:** ADHD traits, anorexia nervosa, autistic traits, familial high‐risk, psychiatric morbidity, psychopathology

## Abstract

**Objective:**

Psychiatric comorbidity frequently presents in anorexia nervosa (AN). Yet, the premorbid mental health status is relatively unknown. The aim of this study was to map out psychiatric morbidity and psychopathology among girls at familial high risk (FHR) of developing AN, thereby detecting possible underlying vulnerabilities preceding the disease.

**Method:**

Twenty‐eight daughters of mothers with a history of AN (FHR daughters), aged 6–12, along with 42 comparison daughters without FHR for AN (COMP daughters), were recruited. The mothers completed a battery of parental diagnostic interviews and screening questionnaires regarding psychiatric diagnoses and psychological traits in their daughters, including the Development and Well‐Being Assessment (DAWBA) and the Strengths and Difficulties Questionnaire (SDQ).

**Results:**

In comparison to the COMP daughters, the FHR daughters had 27 percentage points higher absolute risk of meeting criteria for ≥ 1 psychiatric diagnoses (32.1% versus 4.8%; unadjusted *p* = 0.007; adjusted *p* = 0.035), and 21 percentage points higher risk of ≥ 2 psychiatric diagnoses (unadjusted *p* = 0.007; adjusted *p* = 0.035) according to the DAWBA and/or as established by the Child and Adolescent Psychiatry clinics. Among the FHR daughters, anxiety disorders and autism were the most common diagnoses. In addition, the FHR daughters scored significantly higher on the Emotional symptoms subscale of the SDQ (*p* = 0.03).

**Discussion:**

Increased risks of psychiatric diagnoses and elevated emotional problems were found among girls at FHR for AN. The results may therefore suggest psychiatric morbidity to be a potential risk factor for developing AN.

## Summary

- Young girls at familial high risk (FHR) of developing anorexia nervosa (AN) had almost 30% higher absolute risk of meeting criteria for at least one psychiatric disorder, most commonly anxiety disorders and autism, in contrast with a low‐risk comparison group.
- Based on SDQ data, emotional symptoms were significantly more frequent among the FHR girls compared with the comparison girls.
- Previous research has demonstrated both clinical and genetic associations between AN and anxiety disorders. The higher prevalence of anxiety disorders observed in the FHR girls may therefore represent an early manifestation of vulnerability to AN.

## 1 Introduction

Anorexia nervosa (AN) is a psychiatric disorder characterized by severe underweight, an intense fear of weight gain, and a distorted body image (American Psychiatric Association 2013). The lifetime prevalence of AN in women is estimated up to 3.6% (van Eeden et al. 2021). Among eating disorders (EDs), AN has the highest mortality rate (Arcelus et al. 2011). Treatment options for AN are predominantly psychotherapeutic, and for adolescents, family‐based treatment is the first choice (NICE 2017). A positive outcome is largely dictated by early change, that is, weight gain within the first weeks of treatment (Le Grange et al. 2014). Detecting AN at an early stage is therefore imperative for remission. Even though AN is a severe and well‐studied psychiatric disorder, the knowledge regarding the initial, or premorbid stages is still very limited. As a result, little is known about potential psychopathological vulnerabilities preceding the AN onset.

### 1.1 Comorbidity in Acute Stage AN

Extensive research on the acute and recovered stages of AN shows a strong association with psychiatric comorbidity in adults (Treasure et al. 2015). In adolescents, research is scarcer. However, approximately 50% of adolescents with AN have at least one psychiatric comorbidity, affective and anxiety disorders being the most common (Bühren et al. 2014; Mairhofer et al. 2021; Råstam 1992; Trainor et al. 2020). Posttreatment, comorbidity frequencies decrease. However, the decline appears to differ between diagnoses, showing anxiety disorders to be more persistent than depression (Dobrescu et al. 2020; Trainor et al. 2020). It has been suggested that depression emerges post AN debut, while anxiety disorders tend to exist prior to the AN onset. This may partly explain why treatment is associated with greater improvement in depressive symptoms compared to anxiety symptoms (Hughes 2012).

Previous research supports elevated autistic traits in adults with AN compared with healthy controls (Anckarsäter et al. 2012; Nielsen et al. 2015; Nilsson et al. 1999; Tchanturia et al. 2013), and comorbid autism in AN is associated with greater ED severity (Zhang et al. 2022). Although to a lesser extent, elevated autistic traits have been reported among adolescents with AN (Anckarsäter et al. 2012; Baron‐Cohen et al. 2013; Nielsen et al. 2015; Nilsson et al. 1999; Rhind et al. 2014), and evidence of increased autistic traits during the acute stages of AN has been reported (Nuyttens et al. 2024). A longitudinal study of teenage‐onset AN reported reduced, but remaining autistic traits after the recovery of AN in 25% of the sample (Nuyttens et al. 2024). In contrast, a prospective twin cohort study found no overrepresentation of autistic traits in children with later onset AN (Dinkler et al. 2021). Regarding other neuropsychiatric disorders, comorbid attention‐deficit/hyperactivity disorder (ADHD) has previously been more closely associated with ED subtypes comprising binge‐purging behaviors (Fernández‐Aranda et al. 2013; Wentz et al. 2005). However, a relatively recent review reported the risk of ADHD as two‐folded across all EDs and age groups (Nazar et al. 2016).

Another psychological trait associated with AN is perfectionism. Specifically, the self‐oriented dimension, defined as a person's inner motivation and strivings for perfect achievements (Hewitt and Flett 1991). Self‐oriented perfectionism is more prominent in, and specific to, adolescents with AN and bulimia nervosa (BN), in contrast to individuals with anxiety disorders and depression, as well as healthy controls (Castro‐Fornieles et al. 2007). Perfectionism can also be subdivided into the maladaptive form (high standards and high self‐criticism) and adaptive form (high standards without self‐criticism). A meta‐analysis found support for maladaptive perfectionism to be significantly higher in AN compared with other psychiatric diagnoses, and in contrast with a nonclinical group (Dahlenburg et al. 2019).

Maladaptive eating patterns in childhood have been associated with higher levels of AN symptoms in adolescence (Marchi and Cohen 1990). Avoidant/restrictive food intake disorder (ARFID) was formally added as a separate diagnosis among the Feeding and Eating disorders section when the fifth version of the Diagnostic and Statistical Manual of Mental Disorders (DSM‐5) was launched (American Psychiatric Association 2013). Similar to AN, ARFID is characterized by food avoidance leading to insufficient energy intake and growth deficiency (Zimmerman and Fisher 2017). In a longitudinal study of children with ARFID followed between the ages 2 and 11, almost two thirds of the sample showed moderate to severe malnutrition at the 11‐year‐old assessment. Parallelly, the rate of emotional and behavioral problems increased, especially among the girls. This was highlighted as a potential risk for transit into other EDs as the children approached adolescence (Lucarelli et al. 2018).

### 1.2 Comorbidity and Association With Intelligence Quotient (IQ) in AN

The IQ in both adults and adolescents with AN is indicated to be within normal range relative to the general population (Kjaersdam Telléus et al. 2015; Weider et al. 2016). However, the relationship between IQ and psychiatric comorbidities in AN is relatively unknown. Outside the ED research field, a large population‐based cohort study of young children with intellectual disability has shown higher rates of psychopathology compared to typically developing children (Emerson et al. 2010). Further, a Dutch population‐based twin study of 7‐year‐olds reported a negative correlation between IQ and childhood psychopathology, that is, the lower the IQ the higher the risk of psychopatology (Bruins et al. 2024).

### 1.3 Psychiatric Symptoms in First‐Degree Relatives of Individuals With AN

The emergent pattern of co‐existing anxiety, depression, neuropsychiatric disorders, and perfectionistic traits in AN highlights the issue of “state or trait.” Long‐term follow‐up after remission from AN in adolescents indicates persistent elevations in depressive, anxiety, and obsessive‐compulsive traits (Dobrescu et al. 2020; Holtkamp et al. 2005). Possibly, these features were already present before the AN debut, thus being potential risk factors for AN development. However, research into premorbid or early stages of AN is still limited. To close the knowledge gap, the familial high‐risk (FHR) method, focusing on first‐degree relatives of individuals with AN, could be applied. FHR research allows for the specific targeting of high‐risk individuals to examine early characteristics of risk that are considered relevant for a specific disorder. Studies employing the FHR method have been carried out since the 1950s, and have more commonly been used for other psychiatric disorders with high genetic load, such as schizophrenia and bipolar disorder (Sandstrom et al. 2019). The FHR method takes advantage of the relatively strong heritability in AN, at least 50% (Sullivan et al. 2018), as well as the increased rate in women (Silén and Keski‐Rahkonen 2022). As such, the highest familial risk in AN has been identified among female offspring of women with AN (Steinhausen et al. 2015). To date, studies on psychiatric morbidity in offspring of mothers with AN have been scarce, mostly based on population samples and/or including children of mothers with a history of broad‐spectrum EDs (Martini et al. 2020; Pappaianni et al. 2023, 2025). A previous publication showed a higher prevalence of psychiatric disorders in offspring (aged 2–17) of mothers with adolescent‐onset AN compared to healthy controls (Dobrescu et al. 2024). Further, a longitudinal population‐based study found higher odds of emotional difficulties in 7‐year‐old children whose mothers had AN or BN during pregnancy, compared with controls (Barona et al. 2016). A similar study found higher odds of psychiatric disorders in offspring of mothers with EDs at ages 7, 10, and 13, as well as two‐folded increased odds for emotional disorders, especially anxiety, at ages 7 and 13 (Micali et al. 2014).

### 1.4 Aims

Using the FHR‐method, the aim of this study was to examine whether psychiatric morbidities occur at a higher rate in daughters of mothers with previous or current AN, compared with daughters of unafflicted mothers. The primary study outcome was the occurrence of at least one psychiatric diagnosis. Secondary outcomes were the presence of multiple psychiatric diagnoses and levels of neuropsychiatric traits and psychopathological symptoms, that is, perfectionism, as well as emotional and behavioral problems. In addition, an exploratory association between the frequency of psychiatric diagnoses and IQ was sought. We hypothesized that psychiatric morbidities would be more frequent in daughters at FHR for AN compared with their low‐risk counterparts, representive of an early psychopathological vulnerability increasing the risk of developing AN.

## 2 Method

### 2.1 Participants

#### 2.1.1 FHR Daughters

Inclusion criteria for the *FHR daughters* were (a) biological daughters of women with previous or current AN and (b) between 6 and 12 years of age. The age restrictions were established to avoid children in puberty, and thus the elevated risk of AN debut. Exlusion criterion was a current or previous AN diagnosis among the daughters. No participants were excluded due to this criterion. The FHR daughters comprised 28 individuals, all recruited via their mothers. The majority of participants (*N* = 22) were identified through ED services within the Region Västra Götaland (a county located in the southwest of Sweden with approximately 1,750,000 inhabitants). The remainder were self‐referred via the Swedish patient association for EDs (Swedish: “Frisk & Fri”) (*N* = 6). The mothers were recruited along with their daughters. Since the FHR daughters comprised six pairs of siblings, 22 mothers were included.

#### 2.1.2 Comparison Daughters

A low‐risk group, matched for age and gender, the *comparison (COMP) daughters* was recruited, comprising 42 daughters whose mothers had no history of AN, BN or binge‐eating disorder. The mothers were recruited along with their children. The COMP daughters included one pair of siblings; thus 41 mothers participated. The participants were recruited through different sources, including advertisements in local newspapers in Gothenburg, Sweden, and surrounding municipalities. Bill‐posting in public spaces and digital advertisement at the Region Västra Götaland website were also used. Finally, employees from two clinics at the Sahlgrenska University hospital in Gothenburg were informed about the study via e‐mail. A detailed outline of participants' and mothers' demographics is displayed in Table 1.

**TABLE 1:** Baseline characteristics of participants and participants' biological mothers.

Offspring characteristics			FHR daughters *N* = 28^a^	COMP daughters *N* = 42^b^
Age at examination (years)		*M* (SD)	8.9 (2.3)	8.9 (1.9)
BMI SDS^c^		*M* (SD)	0.4 (1.4)	−0.1 (1.1)
WISC‐V full scale IQ (scaled score)^d^		*M* (SD)	98.2 (11.0)	107.4 (13.0)
Pubertal stage (tanner)	1	*N* (%)	19 (68)	27 (64)
	2	*N* (%)	5 (18)	11 (26)
	3	*N* (%)	3 (11)	4 (10)
	4	*N* (%)	1 (4)	0
Menarche status	No	*N* (%)	26 (93)	42 (100)
	Yes	*N* (%)	2 (7)	0
Maternal characteristics			*N* = 22	*N* = 41
Age at examination (years)		*M* (SD)	41.2 (6.5)	42.1 (4.5)
Education	No university degree	*N* (%)	8 (36)	5 (12)
	University degree	*N* (%)	14 (64)	36 (88)
Employment past 6 months	No employment^e^	*N* (%)	10 (45)	2 (5)
	Part‐time employment^f^	*N* (%)	4 (18)	2 (5)
	Full‐time employment^g^	*N* (%)	8 (36)	37 (90)
Duration all AN (years)		*M* (SD)	12.7 (10.8)	0.0 (0.0)
Current AN	No	*N* (%)	10 (45)	41 (100)
	Yes	*N* (%)	12 (55)	0

*Note:* Descriptive data are presented using means, standard deviations for numeric variables, and as counts and percentages for categorical variables.

Abbreviations: AN, anorexia nervosa; BMI SDS, body mass index standard deviation score; COMP, comparison; FHR, familial high‐risk; IQ, intelligence quotient; *M*, mean; *N*, number; SD, standard deviation; WISC‐V, Wechsler intelligence scale for children—5th edition.

^a^ The sample includes six pairs of siblings.

^b^ The sample includes one pair of siblings.

^c^ BMI SDS was based on age, weight and height at examination. For two FHR daughters, BMI SDS calculations were based on an examination performed approximately 1 year earlier. One FHR daughter declined to have her weight measured; calculations were therefore based on the mother's estimate of the participant's weight and height.

^d^ Full scale IQ was obtained for all participants except three FHR daughters who declined neuropsychological testing.

^e^ No employment: Unemployed, sick leave, sickness benefit.

^f^ Part‐time employment: Refers to mothers whose occupation varied between “No employment” and “Full‐time employment” during the time period in question.

^g^ Full‐time employment: Employed, student and/or parental leave.

### 2.2 Procedure

This study is part of an overarching FHR research project. Apart from the results of parental interviews and questionnaires presented here, various examinations and components of data collection were performed across two study visits. A previous publication gives a detailed description of all study examinations including the results from the neuropsychological test battery (Dahlin et al. 2024).

To confirm a previous or current AN diagnosis in the mothers of the FHR daughters, the ED module of the Structured Clinical Interview for the *Diagnostic and Statistical Manual of Mental Disorders*, Fourth edition (SCID‐IV) was used (First et al. 1997). A supplement of ED criteria according to the fifth edition of the DSM‐5 (American Psychiatric Association 2013) was added. The mothers of the COMP daughters were administered the interview to rule out any ED.

The study was mainly conducted during the COVID‐19 pandemic, which partly affected and delayed the examination procedures. Data collection began in October 2020 after permission to examine healthy individuals was granted by the hospital management group. All examinations performed during the pandemic had to adhere to the current restrictions. The data collection was finished in June 2023.

### 2.3 Instruments

To assess psychiatric morbidity among the FHR and COMP daughters, a computerized parental interview, as well as a battery of digitally administered screening instruments, was used. Both the interview and all screening instruments were completed by the AN and COMP mothers. Due to restrictions stated in the ethical permit, no interviews or screening instruments were completed by the daughters. For two COMP daughters, the child's second caregiver was used as informant. The parental assessments were administered and supervised by the last author (E.W.).

#### 2.3.1 Parental Interview

The computerized Swedish version (www.dawba.net) of the Development and Well‐Being Assessment (DAWBA) parental interview was used to assess the prevalence of psychiatric diagnoses among the FHR and COMP daughters (Goodman et al. 2000). The DAWBA includes both closed and open‐ended questions regarding child and adolescent psychopathology according to the DSM‐5 (American Psychiatric Association 2013). The DAWBA enquires about presence, severity, and duration of symptoms within several psychopathological domains. The domains included in this study were the Social Aptitudes Scale (SAS), (R) Development section, (A) Separation anxiety, (B) Fears of specific things or situations, (C) Fear of social situations, (D) Panic attacks and agoraphobia, (E) Posttraumatic stress, (F) Compulsions and obsessions, (G) Generalized anxiety, (H) Depression, and (P) Dieting, weight and body shape. Based on the responses, the program generates provisional diagnoses. All DAWBA interviews were clinically rated by a psychiatrist, the last author (E.W.), who was blind to group status and ultimately decided on the diagnoses. Data on pre‐existing diagnoses from the Child and Adolescent Psychiatry (CAP) clinics was collected through parental report.

#### 2.3.2 Digital Parental Screening Instruments

The parent‐reported Attention Deficit Hyperactivity Disorder—Rating scale (ADHD‐RS) IV (DuPaul et al. 1998) was administered to the mothers to assess ADHD symptoms among the FHR and COMP daughters. A Danish normative evaluation showed high internal validity for use of the ADHD‐RS in 3–17‐year‐olds. The ADHD‐RS is an 18‐item scale (0–3 points per item; maximum score 54). Nine items assess hyperactivity/impulsivity and nine assess inattention. Scores were calculated by summing subscales and the total ADHD‐RS score. A “screen‐positive” approach was applied, defining a subscale as positive if six or more items scored ≥ 2 points, as per the DSM‐5 diagnostic criteria (American Psychiatric Association 2013).

The Autism Spectrum Screening Questionnaire (ASSQ) was used to assess autistic traits through 27 items (each scored 0–2) (Ehlers et al. 1999). The ASSQ was developed for school‐aged children and can be completed by parents or teachers. In a Norweigan total population sample validation of 7–9‐year old children, the ASSQ was found suitable for autism screen in the general population (Posserud et al. 2009). The total ASSQ score (maximum 54) was calculated, and a screen‐positive approach was applied. The participants were considered screen positive if the total score was ≥ 19 points, as stipulated in a validation study of 6–17‐year‐olds (Ehlers et al. 1999).

To identify individuals screening positive for ARFID, a DSM‐5–based checklist was used (American Psychiatric Association 2013).

Perfectionistic traits were assessed using the Perfectionism subscale of the Swedish version of the Eating Disorder Inventory for Children (EDI‐C) (Garner 1991). The EDI‐C is suitable for screening purposes in preadolescents and adolescents according to a Swedish evaluation of 10–18‐year‐olds, girls especially (Thurfjell et al. 2004). The Perfectionism subscale includes six items (maximum score: 18) and was originally designed for self‐report. The subscale has a proposed cut‐off score of 4.10 in adolescent girls with EDs and 2.59 in nonclinical samples (Thurfjell et al. 2003). As ethical approval for the present study prohibited children from completing questionnaires, permission from the publisher was obtained to adapt the instructions for parent‐report (i.e., “For each item, choose the answer best describing *your child's* way of thinking.”).

The Swedish version of the Strengths and Difficulties Questionnaire (SDQ) was used to assess emotional and behavioral problems (Goodman 1997). The SDQ addresses five areas: Emotional problems, Conduct problems, Hyperactivity/inattention, Peer relationship problems, and Prosocial behavior. The parental version consists of 25 items, has good psychometric properties, and is suitable for use in children aged 4–17 (Smedje et al. 1999). By summing the scores from all subscales except the prosocial scale, the SDQ generates a Total difficulties score. The proposed cut‐off scores from a Swedish validation study were applied: Total difficulties ≥ 14; Emotional symptoms ≥ 5; Conduct problems ≥ 4; Hyperactivity ≥ 6; Peer problems ≥ 5; Prosocial behavior ≤ 6 (Malmberg et al. 2003).

#### 2.3.3 Neuropsychological Tests

The Full‐Scale Intelligence quotient (FSIQ) from the Wechsler Intelligence Scale for Children—Fifth Edition (WISC‐V) (Wechsler 2014) was used to explore correlations between IQ and the presence of psychiatric diagnoses. The FSIQ was calculated from all 10 primary subtests of the WISC‐V, and the variable was obtained from a previously reported assessment of neuropsychological profile (Dahlin et al. 2024).

### 2.4 Statistical Analyses

Baseline characteristics of daughters and mothers were summarized using means and standard deviations (SDs) for continuous variables and counts with percentages for categorical variables; no formal baseline hypothesis testing was undertaken. Descriptive summaries are shown in Table 1.

The primary outcome was the presence (binary) of ≥ 1 psychiatric diagnoses according to the DAWBA and/or CAP Clinics. Secondary binary outcomes included: ≥ 2 DAWBA/CAP diagnoses; at least one diagnosis according to DAWBA, CAP, and/or screen‐positive results for ADHD‐RS, ASSQ, and ARFID; at least two such diagnoses (DAWBA/CAP or screen‐positive results); screen‐positive for ADHD (combined, inattentive, or hyperactive/impulsive form); screen‐positive for ARFID; and screen‐positive for ASSQ. For all binary outcomes, crude risk differences (RDs) between groups were estimated, and statistical significance was assessed using the Fisher–Pitman nonparametric permutation test for differences in proportions. Corresponding 95% confidence intervals (CIs) were obtained by test inversion. To control for potential inflation of Type I error due to multiple comparisons, *p*‐values were additionally adjusted using the Benjamini–Hochberg false discovery rate (FDR) procedure, controlling the FDR at the 5% level. Two screen‐positive cases of ARFID were detected among the COMP daughters. To rule out potential sampling bias (i.e., individuals participating in the study due to preexisting food‐related issues), sensitivity analyses excluding these individuals were performed.

Continuous questionnaire outcomes (ADHD‐RS, SDQ, ASSQ, and EDI‐C Perfectionism) were analyzed with linear mixed‐effects models including a random intercept for family to account for clustering of siblings and correlations between daughters of the same mother. Least‐squares means (LS‐means) were reported by group, together with unadjusted and adjusted mean differences with 95% CIs and two‐sided *p*‐values. Adjusted models included the daughter's age and FSIQ and the mother's age and education as covariates. To quantify standardized effect sizes, Cohen's *d* was calculated for each outcome as the mean difference divided by the marginal pooled SD derived from the mixed model. The marginal SD was defined as the square root of the sum of the variance of the random family effect and the residual variance. Both unadjusted and adjusted d values (with 95% CI) are presented alongside the mean differences.

Logistic regression models were fitted within the FHR daughters' group to examine associations between FSIQ (continuous) and the presence of psychiatric diagnoses. Separate models were run for ≥ 1 DAWBA and/or CAP diagnoses, and ≥ 1 diagnoses according to DAWBA, CAP, and/or screen‐positive results for ADHD‐RS, ASSQ, and ARFID. Results were expressed as odds ratios (ORs) with 95% CI per one‐point increase in FSIQ and two‐sided *p*‐values.

All tests were two‐sided with *α* = 0.05. Analyses were performed using SAS/STAT Software, version 9.4 (SAS Institute Inc., Cary, NC, USA), and IBM SPSS Statistics, version 29 (IBM Corp., Armonk, NY, USA).

### 2.5 Ethics Approval

The study was approved by the Swedish Ethical Review Authority on February 7, 2020 (Dnr: 2019‐05669). Written informed consent was obtained from all participants and their caregivers before the examinations.

## 3 Results

Demographic data on the daughters and their mothers are presented in Table 1.

### 3.1 Prevalence of Psychiatric Diagnoses

As shown in Table 2, the FHR daughters had 27 percentage points higher risk of meeting the criteria for one or more psychiatric diagnoses according to the DAWBA and/or CAP (32.1% vs. 4.8%). The RD was statistically significant (95% CI 0.11–0.44; unadjusted *p* = 0.007; FDR‐adjusted *p* = 0.035). The risk of two or more DAWBA/CAP diagnoses was 21 percentage points higher among the FHR daughters (21.4% vs. 0%), thus yielding a statistically significant RD (95% CI 0.07–0.33; unadjusted *p* = 0.007; FDR‐adjusted *p* = 0.035). Similarly, ≥ 1 diagnoses according to any source (DAWBA, CAP and/or screen‐positive results for ADHD‐RS, ASSQ and/or ARFID) were more common among the FHR daughters (35.7% vs. 11.9%; unadjusted *p* = 0.04; adjusted *p* = 0.10), as were ≥ 2 diagnoses according to the same sources (25.0% vs. 2.4%; unadjusted *p* = 0.014; adjusted *p* = 0.045). No other binary outcomes remained significant after FDR correction (Table 2). Table 3 shows a detailed overview of diagnoses. A subgroup analysis of the FHR daughters, comparing those with and without current maternal AN, revealed no significant differences in any binary outcomes (Table S1).

**TABLE 2:** Primary and secondary analysis for binary outcomes in daughters at familial high risk of anorexia nervosa versus a low‐risk comparison group.

	FHR daughters (*N* = 28)	COMP daughters (*N* = 42)	Risk difference (95% CI)	*p*	Adjusted *p* ^a^
Primary outcome					
≥ 1 DAWBA and/or CAP diagnosis	9 (32.1%)	2 (4.8%)	0.27 (0.11, 0.44)	0.007	0.035
Secondary outcomes					
≥ 2 DAWBA and/or CAP diagnosis	6 (21.4%)	0 (0.0%)	0.21 (0.07, 0.33)	0.007	0.035
≥ 1 DAWBA, CAP, ADHD−RS, ASSQ and/or ARFID diagnoses	10 (35.7%)	5 (11.9%)	0.24 (0.06, 0.43)	0.041	0.10
≥ 2 DAWBA, CAP, ADHD−RS, ASSQ and/or ARFID diagnoses	7 (25.0%)	1 (2.4%)	0.23 (0.07, 0.37)	0.014	0.045
Screen−positive for ARFID	1 (3.6%)	2 (4.8%)	−0.01 (−0.12, 0.07)	1.00	1.00
Screen−positive for ADHD combined form	1 (3.6%)	1 (2.4%)	0.01 (−0.07, 0.07)	1.00	1.00
Screen−positive for ADHD inattentive form	2 (7.1%)	1 (2.4%)	0.05 (−0.06, 0.13)	0.69	1.00
Screen−positive for ADHD hyperactive/impulsive form	1 (3.6%)	0 (0.0%)	0.04 (0.00, 0.07)	0.80	1.00
Screen−positive for ADHD (any form)	4 (14.3%)	2 (4.8%)	0.10 (−0.06, 0.22)	0.33	0.59
Screen−positive for autism	0 (0.0%)	0 (0.0%)	0.00 (NA)	1.00	1.00

*Note:* Values are presented as counts and percentages within the FHR and COMP daughters' groups. Risk differences represent the differences in proportions between groups. Group comparisons were performed using the Fisher–Pitman nonparametric permutation test for differences in proportions. Corresponding confidence intervals were obtained by test inversion.

Abbreviations: ADHD, attention‐deficit/hyperactivity disorder; ADHD‐RS, ADHD‐rating scale; ARFID, avoidant/restrictive food intake disorder; ASSQ, autism spectrum screening questionnaire; CAP, child and adolescent psychiatry; CI, confidence interval; COMP, comparison; DAWBA, development and well−being Assessment; FHR, familial high‐risk; NA, not available (confidence interval not estimable).

^a^ Adjusted for multiple testing using the Benjamini–Hochberg false discovery rate (FDR) procedure.

**TABLE 3:** Psychiatric disorders in daughters at familial high risk of anorexia nervosa and in a low‐risk comparison group.

Current psychiatric disorders	FHR daughters (*N* = 28)	COMP daughters (*N* = 42)
	*N* (%)	*N* (%)
≥ 1 psychiatric diagnoses^a^	9 (32.1)	2 (4.8)
Anxiety disorders	7 (25.0)	1 (2.4)
Mood disorders	0 (0.0)	1 (2.4)
Obsessive‐compulsive disorders	1 (3.6)	0 (0.0)
Autism^b^	4 (14.3)	0 (0.0)
Attention‐deficit/hyperactivity disorder	1 (3.6)	0 (0.0)
≥ 2 psychiatric diagnoses^a^	6 (21.4)	0 (0.0)

*Note:* Descriptive data using counts and percentages.

Abbreviations: COMP, comparison; DSM‐5, DSM—5th edition; DSM‐IV, Diagnostic and statistical manual of mental disorders—4th edition; FHR, familial high‐risk; *N*, number.

^a^ According to the Developmental and well‐being assessment (DAWBA) and/or the Child and adolescent psychiatry clinics.

^b^ According to DSM‐IV and/or DSM‐5 criteria.

The corresponding sensitivity analyses, excluding the COMP daughters who were screen‐positive for ARFID, showed a similar pattern of RD for psychiatric diagnoses between the groups (Table S2). In addition, this analysis yielded a significant difference with respect to having ≥ 1 diagnoses according to any source (DAWBA, CAP, and/or screen‐positive results for ADHD‐RS, ASSQ, and/or ARFID), which was significantly more common among the FHR daughters (35.7% vs. 7.5%; adjusted *p* = 0.031) (Table S2).

### 3.2 Neuropsychiatric Traits and Psychopathological Symptoms

The SDQ Emotional Symptoms' score was significantly higher among the FHR daughters (adjusted *p* = 0.03; Table 4), corresponding to a moderate effect size. No other significant adjusted mean differences were observed for the remaining SDQ subscales or the SDQ Total Difficulties score. Further, there were no significant differences between the two groups regarding traits of ADHD, autism, and perfectionism (Table 4). The sensitivity analyses, excluding COMP daughters with ARFID, showed similar results (Table S3).

**TABLE 4:** Secondary analysis: results for continuous parental questionnaires in daughters at familial high risk of anorexia nervosa versus a low‐risk comparison group.

	FHR daughters *N* = 28 LS‐mean (SE)	COMP daughters *N* = 42 LS‐mean (SE)	Unadjusted mean difference (95% CI)	Unadjusted Cohen's *d* (95% CI)	*p*	Adjusted mean difference (95% CI)	Adjusted Cohen's *d* (95% CI)	*p*
ADHD‐RS inattention subscale (0–27)	6.4 (1.1)	4.6 (0.9)	1.9 (−1.0, 4.7)	0.34 (−0.18, 0.85)	0.20	–0.3 (−3.4, 2.9)	−0.05 (−0.65, 0.55)	0.87
ADHD‐RS hyperactivity/impulsivity subscale (0–27)	5.0 (1.2)	4.2 (0.9)	0.9 (−2.1, 3.9)	0.15 (−0.37, 0.67)	0.56	−0.7 (−4.1, 2.7)	−0.13 (−0.74, 0.48)	0.67
ADHD‐RS total score (0–54)	11.5 (2.2)	8.7 (1.7)	2.8 (−2.7, 8.3)	0.26 (−0.26, 0.78)	0.32	−1.0 (−7.1, 5.1)	−0.10 (−0.70, 0.50)	0.74
SDQ emotional symptoms subscale (0–10)	2.9 (0.4)	1.2 (0.3)	1.6 (0.7, 2.6)	0.82 (0.33, 1.31)	0.001	1.3 (0.2, 2.4)	0.65 (0.08, 1.23)	0.03
SDQ conduct subscale (0–10)	1.1 (0.3)	1.1 (0.2)	−0.0 (−0.7, 0.7)	−0.03 (−0.52, 0.45)	0.89	−0.5 (−1.3, 0.4)	−0.31 (−0.92, 0.29)	0.31
SDQ hyperactivity/inattention subscale (0–10)	2.9 (0.5)	2.7 (0.4)	0.2 (−1.2, 1.6)	0.07 (−0.44, 0.59)	0.77	−0.5 (−2.1, 1.1)	−0.19 (−0.79, 0.42)	0.54
SDQ peer relationships problem subscale (0–10)	1.2 (0.3)	0.7 (0.2)	0.6 (−0.2, 1.3)	0.39 (−0.12, 0.91)	0.13	0.5 (−0.3, 1.4)	0.37 (−0.23, 0.97)	0.22
SDQ prosocial behavior subscale (0–10)	8.9 (0.3)	8.6 (0.2)	0.2 (−0.5, 0.9)	0.17 (−0.34, 0.68)	0.51	0.2 (−0.6, 1.1)	0.17 (−0.43, 0.76)	0.58
SDQ total difficulties score (0–40)	8.0 (1.2)	5.8 (0.9)	2.2 (−0.8, 5.2)	0.38 (−0.13, 0.89)	0.14	0.8 (−2.7, 4.3)	0.14 (−0.45, 0.73)	0.64
ASSQ total score (0–54)	3.0 (0.6)	1.7 (0.5)	1.3 (−0.3, 2.9)	0.42 (−0.10, 0.95)	0.11	0.9 (−1.1, 2.8)	0.27 (−0.33, 0.87)	0.37
EDI‐C perfectionism subscale (0–18)	2.9 (0.6)	2.9 (0.5)	−0.1 (−1.6, 1.5)	−0.02 (−0.54, 0.50)	0.94	0.5 (−1.4, 2.3)	0.15 (−0.46, 0.76)	0.62

*Note:* Models: linear mixed‐effects model with random intercept for family; LS‐mean (SE) are least‐squares means and their standard errors by group; Cohen's *d* (unadjusted) = unadjusted LS‐mean difference divided by marginal SD from the unadjusted mixed model; Cohen's *d* (adjusted) = adjusted LS‐mean difference divided by marginal SD from the adjusted mixed model; Marginal SD = sqrt(Var(random intercept for family) + Var(residual)).

Abbreviations: ADHD‐RS, attention‐deficit/hyperactivity disorder—rating scale; ASSQ, autism spectrum screening questionnaire; CI, confidence interval; COMP, comparison; EDI‐C, eating disorder inventory for children; FHR, familial high‐risk; SDQ, strengths and difficulties questionnaire; SE, standard error.

### 3.3 Correlates Between Psychiatric Diagnoses and IQ Among the FHR Daughters

Table 5 summarizes logistic regression models of psychiatric diagnoses on FSIQ among the FHR daughters. The analysis showed no statistically significant association between FSIQ and criteria fulfillment for ≥ 1 DAWBA and/or CAP diagnoses (*p* = 0.19). Using the broader diagnosis definition (DAWBA, CAP and/or screen‐positive results for ADHD‐RS, ASSQ, and/or ARFID) showed a similar, nonsignificant trend (*p* = 0.69).

**TABLE 5:** Exploratory analyses: correlation between psychiatric diagnoses and full‐scale intelligence quotient in daughters at familial high risk of anorexia nervosa.

Outcome	OR (95% CI) per 1‐point increase in FSIQ	*p*
At least one DAWBA and/or CAP diagnosis	0.94 (0.86, 1.03)	0.19
At least one DAWBA, CAP, ADHD‐RS, ASSQ, and/or ARFID diagnosis	0.98 (0.91, 1.07)	0.69

*Note:* Logistic regression models were used with psychiatric diagnosis (yes/no) within the FHR daughters as the dependent variable and full‐scale intelligence quotient as a continuous predictor. Odds ratios (OR), 95% confidence intervals, and *p*‐values are from these models.

Abbreviations: ADHD, attention‐deficit/hyperactivity disorder—rating scale; ARFID, avoidant restrictive food intake disorder; ASSQ, autism spectrum screening questionnaire; CAP, child and adolescent psychiatry; DAWBA, development and well‐being assessment; FHR, familial high‐risk; FSIQ, full‐scale intelligence quotient; OR, odds ratio.

## 4 Discussion

This study aimed to investigate whether psychiatric morbidity is overrepresented among young girls at FHR for AN compared with a low‐risk comparison group. Overall, the results showed a markedly increased risk of psychiatric diagnoses and elevated emotional symptoms among the FHR daughters. The risks remained significantly higher also after correcting for multiple testing. Based on the DAWBA interviews and the diagnoses assigned at the CAP clinics, the absolute risk of at least one psychiatric disorder was nearly 30 percentage points higher among the FHR daughters compared with the COMP daughters. The risk of having at least two such diagnoses was more than 20 percentage points higher for the FHR daughters. When including screen‐positive results for autism, ADHD, and ARFID, along with the DAWBA/CAP diagnoses, the results also showed a significantly higher risk for psychiatric diagnoses among the FHR daughters.

Compared with other FHR studies, using similar diagnostic interviews, psychiatric disorders have been detected in 10%–20% of the children (Dobrescu et al. 2024; Micali et al. 2014; Pappaianni et al. 2025). However, these studies have included population/community‐based samples as well as different age ranges and mixed sexes. The prevalence of AN is dominant in females compared with males, and the risk of debut drastically increases with the onset of puberty in girls (Treasure et al. 2015; van Eeden et al. 2021). To target the group at the highest possible familial risk of developing AN, this study specifically focused on young female FHR offspring. Dobrescu et al. (2024) investigated the offspring (0–25 years old) of mothers with adolescent‐onset AN in a combined population‐based/population‐screened sample. Although using similar instruments compared with the present study, several important differences are found between the two studies. First, the sample in Dobrescu et al. had a wider age range and a higher mean age (14.8 vs. 8.9 in our sample). As such, a significant proportion had already reached and passed the age of peak AN onset, that is, 15 years of age, with 7.2% of the offspring already identified with a lifetime ED diagnosis. While the study by Dobrescu et al. is a very important research contribution, the results mainly speak to the general knowledge of psychopathology among offspring of individuals with a history of AN. In contrast, the design and sample selection of the current study have the properties necessary to provide information specific to the premorbid status and the potential psychiatric risk factors for AN. Second, the sample in Dobrescu et al. was of mixed genders (approximately 50/50 girls to boys). Due to the limited sample size, no gender‐stratified comparisons were performed in their study. In comparison, we selected female offspring only, thereby ensuring the sample was concentrated to the group at the highest possible risk for AN development. An additional difference between the two studies was the recruitment basis. The AN mothers in Dobrescu et al. were part of a community‐/population‐based sample. In contrast, the AN mothers in this study were predominantly recruited from a clinical setting. Related, the total duration of all AN episodes reported among the mothers in Dobrescu et al. was 5.0 years, as compared to 12.7 years in our sample. Therefore, we propose that the FHR daughters in this study stem from a more clinically severe AN heritage. Nevertheless, the results presented by Dobrescu et al. (2024) constitute the most relevant comparison to our findings, and the presence of any current psychiatric disorder was reported to be 18.6% among the AN offspring in their study. The corresponding percentage among our FHR group exceeded 30%, with autism and anxiety disorders being the most frequent diagnoses. Alongside mood disorders, previous research of both clinical and community‐based samples has detected anxiety disorders as frequently occurring in children and adolescents with ongoing AN (Hughes 2012; Trainor et al. 2020). Similar observations were made among children at FHR for EDs in a community‐based sample (Micali et al. 2014). Anxiety disorders have been suggested to precede the AN onset (Hughes 2012), and have proven to be more persistent, remaining in up to 75% of adolescents, post treatment (Trainor et al. 2020). In a 30‐year follow‐up study of adolescent‐onset AN, anxiety disorders were prevalent in 29% of the sample (Dobrescu et al. 2020). A large genome‐wide association study has previously established a significant genetic correlation between AN and anxiety disorders (Watson et al. 2019). Thus, the frequent prevalence of anxiety disorders in our FHR sample further emphazises the relationship between the two conditions.

In contrast, the analyses of continuous results for the screening instruments assessing parent‐rated symptoms of ADHD, autism, perfectionism and psychological health, yielded no significant differences between the groups. The SDQ subscale for emotional problems, was an exception, and was significantly higher among the FHR daughters, although the mean score for both groups was subclinical. This finding mirrors another FHR study for AN, using a birth cohort sample, detecting higher odds of emotional difficulties according to the SDQ in girls with maternal AN at age seven, compared to a low‐risk comparison group (Barona et al. 2016). In contrast, children aged 2–17, whose mothers had adolescent‐onset AN, reported no differences in psychopathology as measured by any SDQ subscale (Dobrescu et al. 2024). The nonexistant between‐group differences for the remainder of parent‐rated instruments could partly be explained by the narrower diagnostic range covered in these questionnaires, compared with the DAWBA/CAP information sources. Autism was, however, covered in both the diagnostic interview (DAWBA) and the parental questionnaire (ASSQ). According to the diagnoses yielded from the DAWBA and CAP, autism was present among almost 15% of the FHR daughters. Remarkably, none of the participants, irrespective of group, reached the ASSQ clinical cut‐off, despite the instrument's good psychometric properties of accurately identifying autism in population‐based samples (Posserud et al. 2009). Most autism screeners, including the ASSQ, have been developed around the male autism phenotype (Kopp and Gillberg 2011), potentially explaining the relatively low scores detected in our, all‐female sample. Further, perfectionistic traits in childhood, coupled with a vulnerability to anxiety, have been suggested to increase the risk of developing AN (Halmi et al. 2012). In this study, no significant difference between the FHR and COMP daughters regarding the EDI‐C Perfectionism scale was found. Interestingly, the mean value in both groups exceeded the suggested subscale cut‐off score for non‐clinical ED‐screening in adolescent girls (cut‐off: 2.59; FHR and COMP daughters: 2.9) (Thurfjell et al. 2003). In comparison, a study evaluating differences in EDI‐C scores among Swedish 10‐year‐old girls reported a mean Perfectionism scale score of 2.4 in a group with self‐reported disordered eating behaviors, thus lower than both groups in our sample (Edlund et al. 1999). In Sweden, there is a lack of validated assessments of perfectionism developed for children, especially parental versions. Although the EDI‐C is an instrument with good discriminant qualities, that is, to correctly identify subjects with EDs from those without (Thurfjell et al. 2003), it cannot be ruled out that the adaption from the original self‐report format into parental administration may have caused an overestimation of perfectionistic traits in both groups.

The COMP daughters in our study presented with relatively low rates of psychiatric disorders according to the DAWBA and CAP (4.8%). In the initial validation of the DAWBA, a community‐based sample of Brittish children and adolescents, aged 5–15 years, reported a rate of diagnosis among 10.6% (Goodman et al. 2000). However, a Swedish study on offspring, aged 5–25 years, of mothers with adolescent‐onset AN, reported current psychiatric disorders among 4.1% of the comparison group (Dobrescu et al. 2024). Further, the mean SDQ Total difficulties score in our COMP group was 5.8. In comparison, a study of parental SDQ normative data for Swedish 10–13‐year‐olds showed a corresponding mean score of 6.1–6.2 (Björnsdotter et al. 2013). Thus, from a cultural perspective, it can be concluded that the rates of both psychiatric disorders and psychopathology in the COMP group are comparable with other Swedish control samples.

### 4.1 Scientific Novelty

The FHR method has also been applied in other severe psychiatric disorders to identify early risk markers. The significance of prospective FHR studies has been highlighted as a means to advance the understanding of underlying psychopathology involved in the development of conditions such as bipolar disorder and schizophrenia (Gregersen et al. 2022). Similar to AN, these studies of FHR offspring have detected increased risk of various psychiatric disorders among the children and adolescents, including affective and anxiety disorders (De la Serna et al. 2021; Gregersen et al. 2022). In addition, a large longitudinal study including offspring of individuals with bipolar disorder, showed early onset ADHD to predict later development of bipolar disorder (Birmaher et al. 2021). Given the recognized importance of the FHR method in uncovering early risk markers, our results, though limited by sample size and single time point, warrant careful consideration. Our sample was completely concentrated to pre‐adolescent female offspring of mothers with a history of AN, thus providing novel insights into the group of utmost elevated FHR for AN. We suspect that the increased prevalence of psychiatric morbidities in childhood, especially anxiety and autism, may constitute underlying vulnerabilities increasing the risk for later development of AN. Previous research on risk factors for EDs has emphasized the importance of continued, thorough examinations of different aspects of psychological functioning in high‐risk individuals during significant developmental phases, that is, adolescence, as these periods constitute a paramount opportunity for early identification and prevention of EDs (Bakalar et al. 2015). Further, the importance of targeting children *absent* of EDs, has been suggested as a necessary mean to identify risk factors manifesting in early childhood. Ideally, this can be achieved using a prospective study design with continuous periodic assessments of the same individuals (Gardner et al. 2000). Consequently, and keeping the comorbidities associated with acute‐stage AN in mind, extra caution should be taken among girls at FHR for AN who exhibit early psychiatric symptoms. Quite possibly, these symptoms could constitute the first indication of an imminent AN development. A follow‐up study will, however, be required in order to establish whether the vulnerability to psychiatric morbidity in individuals at FHR is predictive of AN development.

### 4.2 Strengths and Limitations

This study targeted the group with the highest individual risk of developing AN. Very few previous research projects have focused on this particular group. Further, the participants were examined using a broad range of well‐validated and frequently used diagnostic instruments, including both parent‐rated and clinically based assessments. All maternal AN diagnoses were established through structured diagnostic interview, thus not screening‐based. Additionally, this study was strengthened by the fact that all instruments were completed for all of the participants, providing a complete data set. Finally, corrections were performed to control for multiple testing across the analyses.

The study was limited by the relatively small sample size. For the overarching research project, the target was 50 individuals per group, ensuring sufficient statistical power. Despite the extensive recruitment period, we were unable to identify the targeted number of participants. Consequently, the limited power may have prevented us from detecting clinically meaningful differences between the groups. Nevertheless, we found robust support for our primary outcome regarding the absolute risks of psychiatric diagnoses. Further, this study permitted the participation of more than one daughter per mother, potentially increasing the risk of overly “optimistic” interpretations of the outcomes. Conversely, only allowing one child per mother would have carried an elevated risk of underestimating the prevalence of psychiatric morbidity among daughters at FHR for AN. Therefore, appropriate statistical measures were taken to account for the non‐independence within the data set, that is, the clustering of siblings.

The interpretation of our results should take into account that the recruitment was primarily from clinical settings. Just over half of the FHR daughters resided with a mother who currently suffered from AN, which in turn could have impacted their psychological well‐being. However, in the subgroup analyses, we found no differences between daughters of mothers with current versus previous AN. Among the FHR daughters, the informants were mothers with a self‐experience of mental disorders, possibly more prone, or sensitive to detect psychological problems in their children, in contrast with the comparison mothers. To counter potential bias, all DAWBA interviews were rated by a child psychiatrist, blinded to group status, and complementing information about established CAP diagnoses was retrieved.

This study was mainly based on parental information. The use of teacher‐rated or self‐report questionnaires could have complemented the diagnostic information. However, self‐reporting was not feasible due to the participants' young age. Additionally, several daughters were not yet aware of their mother's psychiatric history, and the Swedish Ethical Review Authority demanded that all data collection concerning psychological functioning should be strictly parent‐based.

Finally, this study provided no information on psychiatric disorders among the AN mothers, except for prevalence of current or past EDs. Further, we did not investigate whether other family members, besides the mothers, had a history of AN. We can therefore not exclude a potential inheritance from the fathers. Although these are limitations, the objective of this study was to perform an extensive evaluation of the daughters' psychiatric health. Previous research has contributed valuable knowledge regarding the comorbid status of adults with AN.

## 5 Conclusions

Young girls at FHR of developing AN had almost 30 percentage points higher absolute risk of psychiatric morbidity, anxiety disorders and autism being the most frequent, in contrast with a low‐risk comparison group. The morbidity rates observed in this study surpassed those reported in earlier FHR research. This difference likely reflects the sample's unique composition, with young female offspring of mothers with AN, predominantly recruited from clinical settings. Mirroring the comorbidity pattern seen in acute stage AN, the FHR daughters displayed high rates of anxiety disorders. Taken together with prior evidence of genetic overlap between AN and anxiety disorders, our findings suggest that early identification of anxiety in those at FHR for AN warrants consideration in future clinical practice.

## Author Contributions

**Kajsa Järvholm:** writing – review and editing, supervision. **Jovanna Dahlgren:** writing – review and editing, supervision. **Karin Dahlin:** conceptualization, methodology, writing – original draft, writing – review and editing, data curation, formal analysis, investigation. **Elisabet Wentz:** conceptualization; methodology; writing – review and editing; data curation; formal analysis; investigation; supervision. [Correction added after first online publication on 05 May 2026: The Author Contributions section has been updated to remove the duplicate attribution of Karin Dahlin.]

## Lived Experience Involvement Statement

No specific efforts were undertaken to involve persons with lived experience in the study design or execution, or in the preparation of this manuscript.

## Funding

The last author of this study received grant support from the Swedish Research Council (2023‐01799) and the Swedish Governmental Funding of Clinical Research (ALF) (ALFGBG‐965750). The first author received a donation from the Märta och Gustaf Ågrens stiftelse (The Märta and Gustaf Ågren Foundation), distributed through Drottning Silvias barn och ungdomssjukhus forskningsfonder (The Queen Silvia Children's Hospital Research Foundation).

## Ethics Statement

The study was approved by the Swedish Ethical Review Authority on February 7, 2020 (Dnr: 2019‐05669).

## Conflicts of Interest

K.J. has received speaker honoraria from Novo Nordisk, unrelated to this work. The reimbursement was directed to her clinical institution. J.D. received speaker honoraria from Novo Nordisk and Nestlé, unrelated to this work. The other authors declare no conflicts of interest.

## Supporting information


**Table S1:** Subgroup analyses within the daughters at familial high risk of anorexia nervosa (maternal anorexia nervosa currently versus no anorexia nervosa currently).
**Table S2:** Sensitivity analyses (excluding comparison daughters with Avoidant/restrictive food intake disorder) for Primary and secondary binary outcomes.
**Table S3:** Sensitivity analyses (excluding comparison daughters with avoidant/restrictive food intake disorder) for results for continuous questionnaire outcomes.

## Data Availability

The data that support the findings of this study are available from the corresponding author upon reasonable request.
