# Supplementary material for: Psychiatric Morbidity Is Overrepresented in Young Girls at High Risk of Developing Anorexia Nervosa
Source: Int J Eat Disord. 2026 Apr 19;59(8):1740–52. doi: 10.1002/eat.70104 (PMC13446241; doi:10.1002/eat.70104)
Supplement: Supplementary file 1 [file EAT-59-1740-s001.docx]

**Supplementary table 1**

*Subgroup analyses within the daughters at familial high risk of anorexia nervosa (maternal anorexia nervosa currently versus no anorexia nervosa currently)*

|  | **Current AN (N = 16)** | **No current AN**  **(N = 12)** | **Risk difference (95% CI)** | ***P*** |
| --- | --- | --- | --- | --- |
| ≥1 DAWBA and/or CAP diagnosis | 7 (43.9%) | 2 (16.7%) | 0.27 (0.00, 0.67) | 0.27 |
| ≥2 DAWBA and/or CAP diagnoses | 4 (25.0%) | 2 (16.7%) | 0.08 (−0.25, 0.43) | 0.96 |
| ≥1 DAWBA, CAP, ADHD−RS, ASSQ and/or ARFID diagnosis | 7 (43.9%) | 3 (25.0%) | 0.19 (−0.17, 0.57) | 0.54 |
| ≥2 DAWBA, CAP, ADHD−RS, ASSQ and/or ARFID diagnoses | 5 (31.3%) | 2 (16.7%) | 0.15 (−0.20, 0.50) | 0.68 |
| Screen−positive for ARFID | 0 (0.0%) | 1 (8.3%) | −0.08 (−0.20, −0.00) | 0.86 |
| Screen−positive for ADHD combined form | 1 (6.3%) | 0 (0.0%) | 0.06 (0.00, 0.20) | 1.00 |
| Screen−positive for ADHD inattentive form | 2 (12.5%) | 0 (0.0%) | 0.13 (0.00, 0.33) | 0.63 |
| Screen−positive for ADHD hyperactive/impulsive form | 0 (0.0%) | 1 (8.3%) | −0.08 (−0.20, −0.00) | 0.86 |
| Screen−positive for ADHD (any form) | 3 (18.8%) | 1 (8.3%) | 0.10 (−0.17, 0.40) | 0.83 |
| Screen−positive for autism | 0 (0.0%) | 0 (0.0%) | 0.0 (NA) | 1.00 |
| *Note:* Values are presented as counts and percentages within each subgroup (maternal AN currently vs no AN currently). Risk differences represent the differences in proportions between groups. Group comparisons were performed using the Fisher–Pitman nonparametric permutation test for differences in proportions. Corresponding confidence intervals were obtained by test inversion. Abbreviations: ADHD, Attention-deficit/hyperactivity disorder; ADHD-RS, ADHD – rating scale; AN, Anorexia nervosa; ARFID, Avoidant/restrictive food intake disorder; ASSQ, autism spectrum screening questionnaire; CAP, Child and adolescent psychiatry; CI, Confidence interval; DAWBA, Development and well-being assessment; NA, Not applicable; RD, Risk difference. | | | | |

**Supplementary table 2**

*Sensitivity analyses (excluding comparison daughters with avoidant/restrictive food intake disorder) for primary and secondary binary outcomes*

|  | **FHR daughters (N = 28)** | **COMP daughters**  **(N = 42)** | **Risk difference (95% CI)** | **P** | **Adjusted *P **** |
| --- | --- | --- | --- | --- | --- |
| **Primary outcome** |  |  |  |  |  |
| ≥1 DAWBA and/or CAP diagnosis | 9 (32.1%) | 2 (5.0%) | 0.27 (0.11, 0.44) | 0.009 | 0.031 |
| **Secondary outcomes** |  |  |  |  |  |
| ≥2 DAWBA and/or CAP diagnoses | 6 (21.4%) | 0 (0.0%) | 0.21 (0.07, 0.33) | 0.006 | 0.031 |
| ≥1 DAWBA, CAP, ADHD-RS, ASSQ and/or ARFID diagnosis | 10 (35.7%) | 3 (7.5%) | 0.28 (0.10, 0.47) | 0.009 | 0.031 |
| ≥2 DAWBA, CAP, ADHD-RS, ASSQ and/or ARFID diagnoses | 7 (25.0%) | 1 (2.5%) | 0.23 (0.07, 0.37) | 0.014 | 0.035 |
| Screen-positive for ARFID | 1 (3.6%) | 0 (0.0%) | 0.04 (0.00, 0.08) | 0.83 | 1.00 |
| Screen-positive for ADHD combined form | 1 (3.6%) | 1 (2.5%) | 0.01 (−0.07, 0.07) | 1.00 | 1.00 |
| Screen-positive for ADHD inattentive form | 2 (7.1%) | 1 (2.5%) | 0.05 (−0.07, 0.13) | 0.74 | 1.00 |
| Screen-positive for ADHD hyperactive/impulsive form | 1 (3.6%) | 0 (0.0%) | 0.04 (0.00, 0.08) | 0.81 | 1.00 |
| Screen-positive for ADHD (any form) | 4 (14.3%) | 2 (5.0%) | 0.09 (−0.06, 0.23) | 0.37 | 0.74 |
| Screen-positive for autism | 0 (0.0%) | 0 (0.0%) | 0.00 (NA) | 1.00 | 1.00 |
| *Note:* Values are presented as counts and percentages within the FHR and COMP daughters’ groups. Risk differences represent the differences in proportions between groups. Group comparisons were performed using the Fisher–Pitman nonparametric permutation test for differences in proportions. Corresponding confidence intervals were obtained by test inversion. *Adjusted for multiple testing using the Benjamini–Hochberg false discovery rate (FDR) procedure.  Abbreviations: ADHD, Attention-deficit/hyperactivity disorder; ADHD-RS, ADHD – rating scale; ARFID, Avoidant/restrictive food intake disorder; ASSQ, Autism spectrum screening questionnaire; CAP, Child and adolescent psychiatry; CI, Confidence interval; COMP, Comparison; DAWBA, Development and well-being assessment; FHR, Familial high risk; NA, Not applicable; RD, Risk difference. | | | | | |

**Supplementary table 3**

*Sensitivity analyses (excluding comparison daughters with avoidant/restrictive food intake disorder) for results for continuous questionnaire outcomes*

|  | **FHR daughters N = 28 LS-Mean (SE)** | **COMP daughters**  **N = 40 LS-Mean (SE)** | **Unadjusted difference (95% CI)** | **Unadjusted Cohen's d (95% CI)** | **P-value** | **Adjusted difference (95% CI)** | **Adjusted Cohen's d (95% CI)** | **P-value** |
| --- | --- | --- | --- | --- | --- | --- | --- | --- |
| ADHD-RS inattention subscale (0−27) | 6.5 (1.2) | 4.8 (0.9) | 1.7 (−1.2,4.6) | 0.30 (−0.22,0.82) | 0.25 | −0.4 (-3.6,2.8) | −0.08 (−0.69,0.52) | 0.79 |
| ADHD-RS hyperactivity/impulsivity subscale (0−27) | 5.0 (1.2) | 4.4 (0.9) | 0.7 (−2.4,3.7) | 0.11 (−0.41,0.64) | 0.67 | −0.9 (−4.3,2.6) | −0.16 (−0.77,0.46) | 0.61 |
| ADHD-RS total score (0−54) | 11.5 (2.2) | 9.1 (1.7) | 2.4 (−3.2,8.0) | 0.22 (−0.30,0.75) | 0.40 | −1.3 (−7.6,4.9) | −0.13 (−0.74,0.48) | 0.67 |
| SDQ Emotional symptoms subscale (0−10) | 2.9 (0.4) | 1.2 (0.3) | 1.6 (0.6,2.6) | 0.81 (0.31,1.30) | 0.002 | 1.2 (0.1,2.4) | 0.63 (0.05,1.21) | 0.03 |
| SDQ Conduct subscale (0−10) | 1.1 (0.3) | 1.1 (0.2) | 0.0 (−0.7,0.7) | 0.02 (−0.48,0.51) | 0.95 | −0.5 (−1.3,0.4) | −0.34 (−0.94,0.27) | 0.27 |
| SDQ Hyperactivity/Inattention subscale (0−10) | 2.9 (0.5) | 2.8 (0.4) | 0.1 (−1.3,1.5) | 0.03 (−0.49,0.56) | 0.90 | −0.6 (−2.2,1.0) | −0.22 (−0.83,0.38) | 0.47 |
| SDQ Peer relationships problem subscale (0−10) | 1.2 (0.3) | 0.7 (0.2) | 0.6 (−0.2,1.3) | 0.40 (−0.12,0.92) | 0.13 | 0.5 (−0.4,1.4) | 0.36 (−0.25,0.96) | 0.24 |
| SDQ Prosocial behaviour subscale (0−10) | 8.9 (0.3) | 8.6 (0.2) | 0.2 (−0.5,0.9) | 0.17 (−0.35,0.68) | 0.52 | 0.3 (−0.6,1.1) | 0.18 (−0.42,0.78) | 0.54 |
| SDQ Total difficulties score (0−40) | 8.0 (1.2) | 5.8 (0.9) | 2.2 (−0.8,5.2) | 0.37 (−0.14,0.89) | 0.15 | 0.7 (−2.8,4.1) | 0.11 (−0.48,0.71) | 0.70 |
| ASSQ total score (0−54) | 3.0 (0.6) | 1.5 (0.5) | 1.4 (−0.2,3.0) | 0.47 (−0.05,1.00) | 0.08 | 0.8 (−1.0,2.7) | 0.27 (−0.34,0.88) | 0.37 |
| EDI-C Perfectionism subscale (0−18) | 2.9 (0.6) | 2.9 (0.5) | 0.0 (−1.5,1.6) | 0.01 (−0.52,0.53) | 0.98 | 0.5 (−1.4,2.3) | 0.15 (−0.46,0.76) | 0.62 |
| *Note:* Models: linear mixed-effects model with random intercept for family. LS-Mean (SE) are least-squares means and their standard errors by group. Cohen's d (unadjusted) = unadjusted LS-mean difference divided by marginal SD from the unadjusted mixed model. Cohen's d (adjusted) = adjusted LS-mean difference divided by marginal SD from the adjusted mixed model. Marginal SD = sqrt(Var(random intercept for family) + Var(residual)). Abbreviations: ADHD-RS, Attention deficit/hyperactivity disorder - rating scale; ASSQ, Autism spectrum screening questionnaire; CI, Confidence interval; COMP, Comparison; EDI-C, Eating disorder inventory for children; FHR, Familial high risk; SDQ, Strengths and difficulties questionnaire; SE, Standard error. | | | | | | | | |
